# Tumor slice culture system to assess drug response of primary breast cancer

**DOI:** 10.1186/s12885-016-2119-2

**Published:** 2016-02-09

**Authors:** Kishan A. T. Naipal, Nicole S. Verkaik, Humberto Sánchez, Carolien H. M. van Deurzen, Michael A. den Bakker, Jan H.J. Hoeijmakers, Roland Kanaar, Maaike P.G. Vreeswijk, Agnes Jager, Dik C. van Gent

**Affiliations:** Department of Genetics, Cancer Genomics Netherlands, Erasmus University Medical Center, PO box 2040, Rotterdam, 3000CA The Netherlands; Department of Pathology, Erasmus University Medical Center, PO box 2040, Rotterdam, 3000CA The Netherlands; Department of Pathology, Maasstad Hospital, Maasstadweg 21, Rotterdam, 3079 DZ The Netherlands; Department of Radiation Oncology, Erasmus University Medical Center, PO box 2040, Rotterdam, 3000CA The Netherlands; Department of Human Genetics, Leiden University Medical Center, P.O. Box 9600, Leiden, 2300 RC The Netherlands; Department of Medical Oncology, Erasmus University Medical Center, PO box 2040, Rotterdam, 3000CA The Netherlands

**Keywords:** Breast cancer, Organotypic tumor tissue slices, Tissue culture method, FAC chemotherapy, Ex vivo sensitivity

## Abstract

**Background:**

The high incidence of breast cancer has sparked the development of novel targeted and personalized therapies. Personalization of cancer treatment requires reliable prediction of chemotherapy responses in individual patients. Effective selection can prevent unnecessary treatment that would mainly result in the unwanted side effects of the therapy. This selection can be facilitated by characterization of individual tumors using robust and specific functional assays, which requires development of powerful ex vivo culture systems and procedures to analyze the response to treatment.

**Methods:**

We optimized culture methods for primary breast tumor samples that allowed propagation of tissue ex vivo. We combined several tissue culture strategies, including defined tissue slicing technology, growth medium optimization and use of a rotating platform to increase nutrient exchange.

**Results:**

We could maintain tissue cultures for at least 7 days without losing tissue morphology, viability or cell proliferation. We also developed methods to determine the cytotoxic response of individual tumors to the chemotherapeutic treatment FAC (5-FU, Adriamycin [Doxorubicin] and Cyclophosphamide). Using this tool we designated tumors as sensitive or resistant and distinguished a clinically proven resistant tumor from other tumors.

**Conclusion:**

This method defines conditions that allow ex vivo testing of individual tumor responses to anti-cancer drugs and therefore might improve personalization of breast cancer treatment.

**Electronic supplementary material:**

The online version of this article (doi:10.1186/s12885-016-2119-2) contains supplementary material, which is available to authorized users.

## Background

Breast cancer (BC) is the most frequently occurring type of malignancy in women and also the leading cause of cancer related deaths among women in high-income countries [1]. BC remains a serious issue in current healthcare, although diagnostic and therapeutic strategies have improved over the past decades. In addition to first line chemotherapeutic treatments, targeted therapies for cancers overexpressing the estrogen, progesterone and Her2 receptor already led to improved patient survival over the past decade [2]. Nevertheless, a subgroup of BC patients either does not respond to first line chemotherapy, or develops resistance. Furthermore, no tailored therapy is currently available for tumors without expression of specific receptors. Therefore, improved personalization strategies for BC treatment are urgently needed.

The most important goal of personalized medicine is to dedicate the most appropriate treatment to the individual patient. This could lead to a situation where the percentage of non-responders and the high proportion of adverse effects of classical chemotherapeutics could be minimized. The success of this approach depends on extensive characterization of individual tumors and their sensitivity to chemotherapy. The majority of preclinical research for treatment efficacy in BC has been performed using established BC cell lines and mouse models. These models are usually not generated from primary BC and resemble only a subset of the diverse types of tumors observed in primary BC. In addition, BC cell lines have been in culture now for decades and have acquired several changes that could affect their biological behavior and therefore they do not faithfully reflect the tumor of origin [3]. Hence, a short-term primary culture derived directly from the tumor is necessary for better characterization and generation of chemotherapy sensitivity profiles in breast tumors from patients.

Various strategies have been applied to generate primary cultures from individual tumors which include: a) 2D culture of dissociated tumor cells, b) 3D spheroid cultures, c) patient derived mouse xenograft (PDX) cultures and d) organotypic tumor slice cultures [4–7]. These strategies all have their own advantages and limitations depending on the specific research purpose.

Tumor dissociation and 2D cultures are suitable for certain tumor types only, because not all primary tumors grow in a monolayer ex vivo and dissociation strategies are very challenging [8, 9]. Moreover, in 2D, tumor architecture is completely lost and this method of culturing causes high selection of tumor cells that grow out [10]. Especially in very heterogeneous cancers, such as BC, tumor cell selection limits the usefulness of this culture option for optimal drug response testing.

Primary tumor cells can also be cultivated ex vivo in 3D in a gelatinous protein mixture, mimicking the extracellular matrix. The generation of 3D tumor spheroid cultures can be applied to more tumor types and tumor cells can be expanded in great numbers for high throughput drug testing. The major disadvantage of this approach is that this expansion of tumor cells takes months of culturing and is therefore not optimal for diagnostic purposes [5].

Another method to reliably assess responses to cytotoxic treatments is the generation of PDX models,. [11, 12] which are generated by implanting pieces of fresh human tumor tissue subcutaneously in immune-deficient mice [7]. However, the successful engraftment rate of breast tumors is less than 25 % and outgrowth of the engraftment takes months. Therefore, this method is not optimal for studying cytotoxic drug responses for personalized cancer treatment.

The organotypic slice method turns out to be ideal for the purpose of short-term primary cultures. It can be applied to most solid tumors and the tissue processing is relatively fast compared to other methods which demand much longer waiting times for ex vivo tumor proliferation [6, 13, 14]. It does not involve selective outgrowth of tumor cells and short-term assays that could predict clinical drug responses can be readily performed making this method in principle ideal for studies on personalized BC treatment [14–16].

Nevertheless, ex vivo assays for personalized treatment based on the tumor tissue slice model are delicate because it is a low throughput assay and methodological developments are challenging [16]. Moreover, tumor heterogeneity requires advanced analytical tools to faithfully categorize tumor responses to drug treatment, especially in the case of BC.

We improved on previously reported organotypic tumor tissue slice methods and optimized it for the ex vivo culture of primary BC. Using reliable markers of cell proliferation and cell death we developed a robust analytical system for breast tumor slices ex vivo. Using these methods we characterized cytotoxicity responses of individual breast tumor slices to chemotherapy.

## Methods

### Collection of tumor tissue

Fresh breast tumor tissue was obtained from BC patients undergoing mastectomy or breast conserving surgery at the Erasmus University Medical Center (Erasmus MC), Havenziekenhuis Rotterdam or Maasstad Hospital Rotterdam, The Netherlands. After resection the tissue was directly transported to the pathology department of the Erasmus MC. After macroscopic investigation and determination of tumor areas for diagnostic purposes by a pathologist, left over tumor tissue was used for research purposes according

to the code of proper secondary use of human tissue in the Netherlands established by the Dutch Federation of Medical Scientific Societies and approved by the local Medical Ethical committees. No informed consent was needed for this study, which has been approved by the Erasmus MC Medical Ethical Committee (number MEC-2011-098). Research samples were kept at 4 °C and transported in specific breast medium (Medium I, Table 1). Specimens were coded anonymously in a way that they were not traceable to the patient by lab workers.

**Table 1:** Overview of medium compositions

Medium I [19]	Medium II [20]	Medium III	Medium IV
- DMEM: HAM's F12 = 2:1 - FCS 2 % - Hydrocortisone 0.3 μg/ml - Insulin 4 μg/ml - Transferrin 4 μg/ml - 3,3´,5 Triiodothyronine 1 ng/ml - EGF 8 ng/ml - Cholera toxin 7 ng/ml - Adenine 0.2 mg/ml - Antibiotics	- RPMI-1640 - FCS 10 % - L-Glutamine 2 nM - Hydrocortisone 5 μg/ml - Insulin 5 μg/ml - Cholera toxin 50 ng/ml - EGF 10 ng/ml - Antibiotics	- RPMI-1640 - FCS 10 % - Antibiotics	- DMEM: HAM's F10 = 1:1 - FCS 10 % - Antibiotics

### Tissue work up, slice preparation and culture

Tumor specimens were subjected to manual and/or automated tissue slicing. Excess fat tissue was discarded using surgical tools and tissue slices of approximately 2 mm thickness were manually generated under sterile conditions. Automated slicing was performed using a Leica VT 1200S Vibratome with slice thickness set at 300 μm, vibration amplitude at 3.0 mm and slicing speed at 0.6 mm/sec. Slicing was performed under semi-sterile conditions; without the use of a flow hood. No contaminations were encountered under these conditions. Slices were cultured within 6 hours after the tumor was removed from the patient. Culturing was performed at 5 % CO_2_ at 37 °C and at atmospheric oxygen levels. Different culture media were tested for quality assessment. Detailed medium compositions are summarized in Table 1. Where indicated, culture dishes were subjected to rotation at 60 rpm using a Stuart SSM1 mini orbital shaker that was placed in the incubator. When indicated, FAC treatment was started directly after slicing the tumor with the indicated concentrations in the culture media. Proliferating cells were labeled using 3 μg/ml EdU (Invitrogen) during the last 2 hours before fixation. Also in case of FAC treatment, EdU was added on the last day during the final 2 hours of incubation. Tumor slices were fixed in 10 % neutral buffered formalin for at least 24 hours at room temperature. Subsequently, tumor slices were embedded in paraffin and 4 μm sections were generated for microscopy analysis.

### Staining protocols

Histological tumor architecture was examined by H&E staining. For immunostaining paraffin sections were deparaffinized in xylene and subsequently hydrated by incubation in decreasing concentrations of ethanol. Target antigen retrieval for Keratin staining was achieved using Citric Acid buffer (2.15 mg/ml) pH 6.0 for 18 min in a microwave at 600 W. Primary antibody anti-PanCytokeratin (AE1/AE3) (Santa Cruz Biotechnology, sc-81714, diluted 1/500) was incubated for 90 min at room temperature and a secondary Alexa Fluor 488 antibody conjugate was used to detect the first antibody. EdU incorporation was visualized using Click-It chemistry (Invitrogen) by incubating samples for 30 min with freshly made Click-It Alexa Fluor 594 cocktail (manufacturers protocol). Samples were mounted using Vectashield mounting medium with DAPI and visualized using a Leica SP5 confocal microscope.

### TUNEL assay

TUNEL assay was performed using In Situ Cell Death Detection Kit (Roche Life Sciences). After deparaffinization and hydration, samples were incubated with Protease K (2 μg/ml) diluted in PBS/ 0.5 % Triton X-100 for 15 min at room temperature. Subsequently, samples were incubated with kit enzyme mix (manufacturers protocol) for 60 min at 37 °C in a humidified environment. After washing with PBS samples were mounted with DAPI.

### Image analysis

From each tumor slice section multiple images were generated using a Leica SP5 confocal microscope. Image size: 512x512 pixels, pixel size ~0.7 μm. For each image field two separate channels were used to detect keratin and EdU signal. Keratin positive area and number of EdU positive cells were semi-automatically determined using a previously described MATLAB (Mathworks) algorithm with minor modifications [17]. In brief, keratin positive areas were detected by first performing a morphological reconstruction that filled dark pixels surrounded by lighter pixels. Then a Sobel edge detection operation with user-defined threshold was applied. Detected regions are filled after opening and closing morphological operations and the number of pixels enclosed in the detected area were used for computing the keratin positive area. Number of EdU positive cells was estimated by first converting the grayscale image in a binary mask with a user-defined threshold and removing objects smaller than 10 pixels from the image. Remaining objects were filled by morphological reconstruction. Finally, the image was eroded with a flat structuring element and objects with more than 10 pixels counted and expressed as EdU positive cells.. Analysis of TUNEL staining was performed using FIJI image analysis software. Separate image channels (image size: 512 x 512 pixels, pixel size ~0.7 μm) for TUNEL and DAPI were automatically thresholded using the Otsu algorithm. In the Coloc2 FIJI plugin, an analysis for co-localization was performed using the Manders co-localization algorithm [18]. Statistical analysis and generation of graphs was performed using Graphpad Prism 6.0.

## Results

### Organotypic tissue slice method

The organotypic tissue slice method starts with the generation of several tissue slices from a fresh tumor specimen after surgical resection. We compared manual slicing, semi-automated slicing (Campden Instruments Ltd.) and automated tissue slicing (Krumdieck MD4000 and Leica Vibratome VT 1200S) in order to optimize the processing and timing of the slice procedure and to maintain tissue morphology during culturing of tumor samples. Manual slicing (using fine surgical tools) and automated tissue slicing using Vibratome VT 1200S (Leica) demanded less tissue processing than semi-automated slicing and automated slicing using Krumdieck MD4000. As this resulted in shorter processing times, we focused on manual and Vibratome VT 1200S mediated tissue slicing.

To investigate whether the vibrating razor blade induced anomalies in tissue morphology, we analyzed general morphology and architecture of individual slices by hematoxilin and eosin (H&E) staining at various times after slicing. No difference was detected between manual tissue slicing and Vibratome VT 1200S slicing, indicating that the vibrating razor blade did not induce additional artefacts (data not shown).

### Optimal tumor slice thickness

**Fig. 1:**
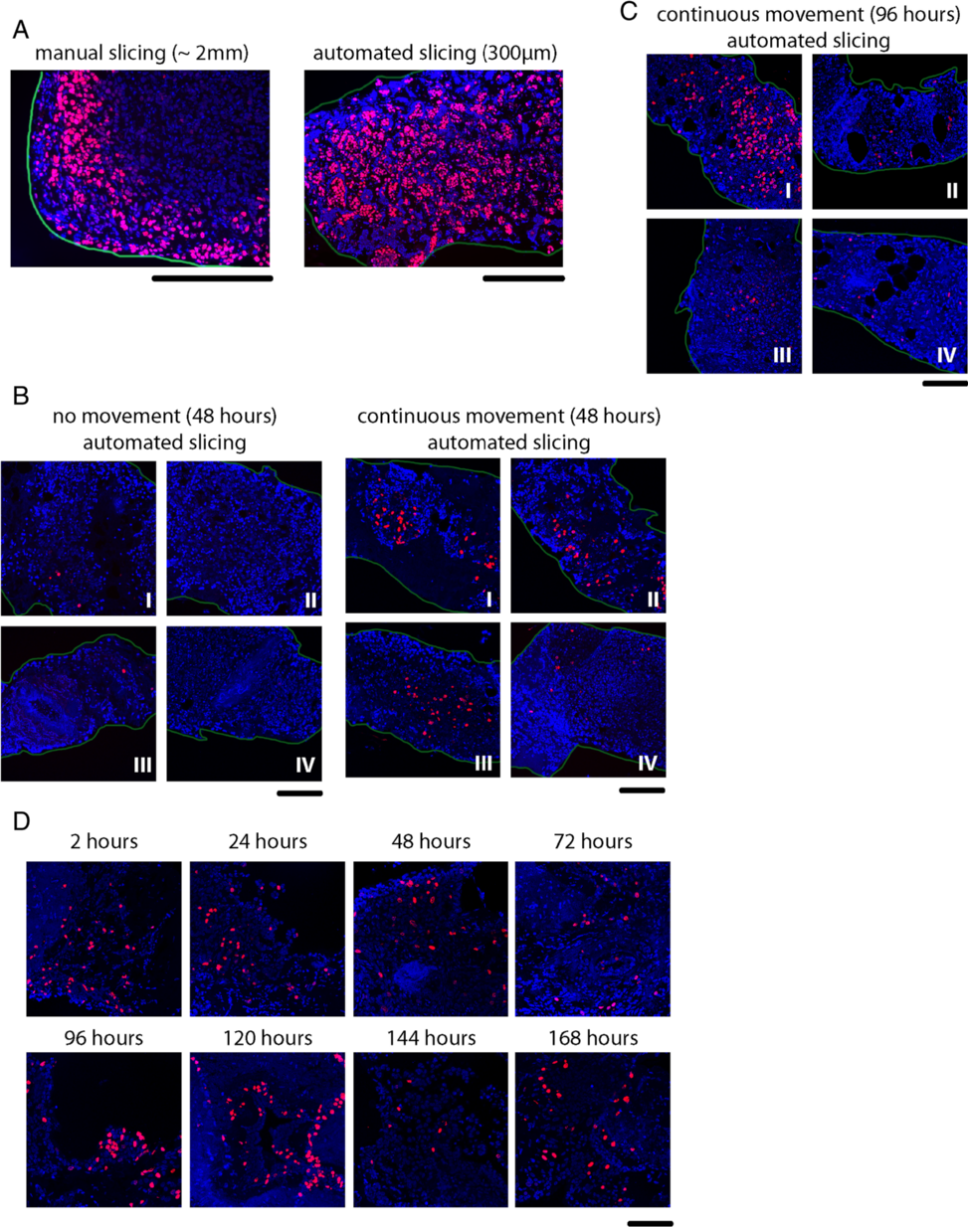
Improvement of organotypic tissue slice viability. **a** Manually sliced tumor slices were incubated for 2 hours in the presence of EU (Ethynyl Uridine) before fixation. During this time penetration of EU is limited to 10–20 cell layers from the edge of the slice. Automatically sliced (300 μm) tumor slices display EU incorporation across the entire depth of the slice within a 2-hour labeling period. **b** Constant orbital movement (60 rpm) significantly increased the number of EdU positive cells after 48 hours incubation compared to static culture conditions. **c** EdU incorporation after 96 hours of culturing under constant movement. **d** Prolonged culture of 300 μm tumor slices from one individual tumor using continuous movement and Medium I. Blue = DAPI, Red = EdU, Green lines indicate the edge of the tumor slice. Scale bars indicate 100 μm

We performed a labeling with the Uracil analog 5-Ethynyl Uridine (EU) to investigate the optimal slice thickness for good penetration of culture medium nutrients in tumor slices. This compound was chosen because EU is incorporated into newly transcribed RNA and transcriptionally active cells are expected to stain positive after a two-hour labeling period. Cells deprived from cell culture nutrients are expected to have less transcription resulting in a lower staining intensity. Manual slicing resulted in slices of approximately 2 mm thickness, with a high level of variability between individual slices. EU labeling of these slices resulted in a gradient staining pattern limited to 10–20 cell layers (approximately 150 μm) from the edge of the slice (Fig. 1a). This indicates that penetration of EU and presumably also culture medium components was suboptimal within a two-hour period.

Automated tissue slicing using Vibratome VT1200 S resulted in slices of precisely defined thickness. Although not frequently observed, soft, mucinous and fibrous tumors could not be processed into very thin slices of less than 500 μm and are therefore less suited for automated tissue slicing (data not shown). Since EU incorporation was detected up to 150 μm from both sides of the tissue sample, we generated standard slices of 300 μm thickness. Indeed, EU labeling of these slices showed optimal incorporation across the entire slice within a two hour labeling period (Fig. 1a). Making thinner slices also had the advantage that more slices could be obtained from an individual tumor.

### Optimal culture medium and culture conditions

To investigate the impact of different culture media on viability of tissue slices we incubated them with four different culture media that have been reported previously for the incubation of breast tumor slices (Medium I-II [19, 20]) and two generally used cell culture media (Medium III-IV; see Table 1). As most classic anti-cancer treatments target rapidly proliferating cells, optimal drug response testing requires maintenance of proliferation rate during ex vivo culture. We investigated the maintenance of proliferative capacity by quantifying EdU (Ethynyl-deoxy Uridine) positive cells after a two-hour labeling period following a previous 24-hour incubation in the four culture media. In manually cut slices we identified low numbers of EdU positive cells, but incubation in medium I showed clearly more EdU positive cells compared to other culture media (Additional file 1: Figure S1A).

Subsequently, we investigated whether culturing under constant movement on an orbital shaker (60 rpm) could maintain replicative potential. Without movement, the slices remained on the bottom of the culture dish during incubation. However, continuous movement caused slices to float in the culture medium, which may promote nutrient exchange between the slice and the medium (Additional file 1: Figure S1B). Indeed, more replicating cells could be detected using continuous movement in all tested media compared to stationary conditions, with the highest number of EdU positive cells achieved with medium I (Additional file 1: Figure S1C). Culture for 48 hours also resulted in more EdU positive cells in the presence of movement for all culture media, although a clear decrease in proliferative capacity was observed compared to samples that were cultured for two hours (Additional file 2: Figure S2). Similar to the EU labeling results, EdU labeling of the manually cut slices showed positively staining cells mainly at the edge of the tissue over a time course of two hours indicating that a proper replicative index cannot be determined for the manually cut tumor slices (Additional file 1: Figure S1 and Additional file 2: Figure S2).

Automated slicing did not maintain replicative potential in the absence of movement (Fig. 1b). However, continuous movement significantly increased the number of EdU positive cells . Automated slicing even resulted in similar numbers of EdU positive cells after 2 and 48 hours of culturing (Fig. 1b and Additional file 2: Figure S2A). The preservation of replicative potential was detected in all different media, although medium IV showed a much lower number of EdU positive cells than the other media (Fig. 1b). A 96-hour incubation under continuous movement with the four different media resulted in the highest number of EdU positive cells in medium I (Fig. 1c).

We conclude that continuous movement during incubation of these tumor slices is absolutely necessary and thinner slices give superior results in case of extended ex vivo incubations. For prolonged culturing, the optimal medium composition is medium I. Using these optimized conditions we could preserve tumor cell proliferation in individual tumor slices ex vivo for at least 7 days after surgical resection (Fig. 1d). Morphologically, no differences were noticed in slices that were fixed at day 0 compared to slices that were fixed at day 7. Also, we did not observe consistent differences in tumor-stroma ratios after prolonged incubations of slices derived from the same tumor (Additional file 3: Figure S3).

### Tissue slice culture method preserves tumor cell proliferation

**Fig. 2:**
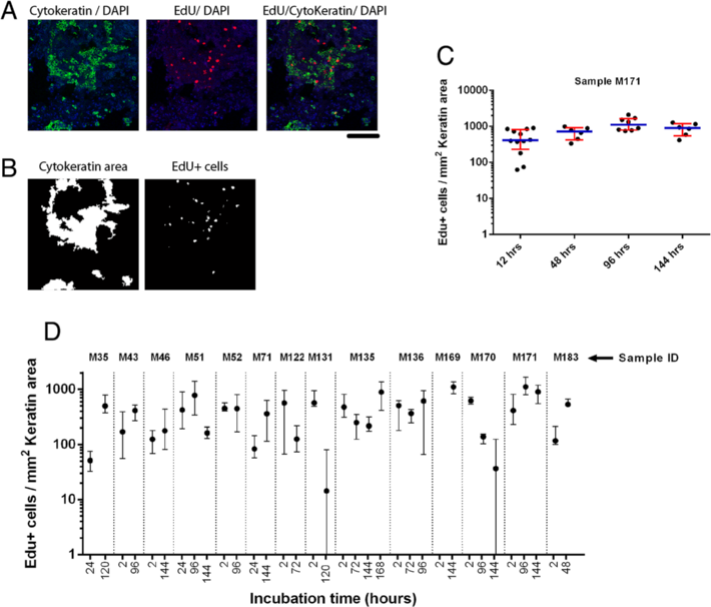
Assessment of proliferation in tumor slices by EdU incorporation. **a** Co-staining for EdU (red), Cytokeratin (green) and DAPI (blue). Scale bar indicates 100 μm. **b** Screenshots of semi-automated measurement of Cytokeratin area and number of EdU positive cells using image analysis software. **c** Example of tumor proliferation after prolonged culture of tumor slices. Multiple image fields were analyzed per tumor slice. Heterogeneity in proliferation is visualized in this graphical representation by interquartile ranges. Each black dot represents one image field. Red bars indicate interquartile range and blue bars represent median values. **d** Proliferation rate of multiple tumors after incubation for up to seven days. Maximum incubation times varied per tumor depending on availability of tumor slices. Black dots indicate median values and error bars represent interquartile range

Standardized analysis of proliferative capacity for individual tumors is difficult to perform due to tumor heterogeneity, both between different tumors and within the same tumor. Tumor-stroma ratios as well as proliferation rates varied widely within tumor slices (Additional file 2: Figure S2C, D). Therefore, we developed an image analysis method that allowed faithful comparison between different tissue slices and individual tumors. We analyzed the number of EdU positive cells per Cytokeratin positive tumor area (Fig. 2a, b). This approach allowed characterization of proliferation in tumor specific areas without being biased by excessive stromal components or heavy infiltration of lymphoid cells, as only Cytokeratin positive areas were monitored. Heterogeneity in proliferation rate was taken into account by randomly imaging multiple image fields per slice (Fig. 2c). This approach revealed that median tissue proliferation rates remained similar after prolonged culture of different tissue slices. However, individual image fields incidentally showed either relatively low or high numbers of EdU positive cells (Fig. 2c). We plotted the interquartile range of EdU positive cells in individual slices and found that these values did not significantly change after prolonged culture of tumor slices, suggesting that the intrinsic proliferative capacity of tumor cells remained constant over time (Fig. 2c, d). We cultured several tumors for up to 7 days and found that the majority of them showed near constant proliferation rates over this time period (Fig. 2d).

### Minimal induction of cell death during prolonged ex vivo incubation

**Fig. 3:**
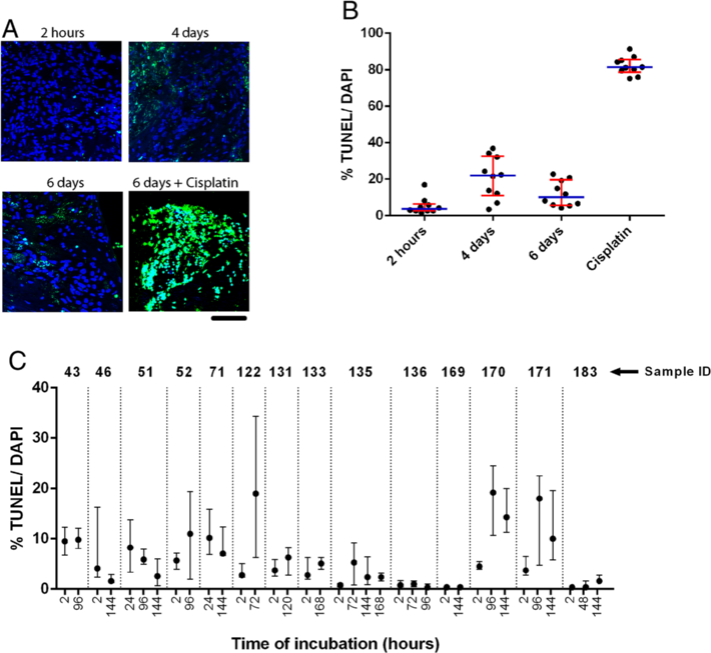
Induction of cell death after prolonged culture of tumor slices is minimal. **a** Representative images of tumor slices from the same tumor displayed similar TUNEL staining intensities at 2 hours, 4 days and 6 days of incubation. Tumor slices incubated with high concentrations (10 μg/ml) of the chemotherapeutic compound Cisplatin revealed massive TUNEL signal. This specific signal was regarded as the positive control for TUNEL signal. Blue = DAPI, Green = TUNEL. Scale bars represent 100 μm. **b** Quantification of TUNEL in different tumor slices. TUNEL signal is variable due to tumor heterogeneity. Each black dot represents one image field. For each image field the percentage of TUNEL-positive DAPI pixels is given. Error bars indicate interquartile range and blue bars represent median values. **c** TUNEL signal was determined for multiple tumors after short and prolonged incubation. Incubation times varied per tumor depending on availability of tumor slices. Black dots indicate median values. Error bars indicate interquartile range

Depending on in vivo tumor necrosis, tissue transportation times and duration of processing, individual tumors might already display a certain level of cell death before being incubated ex vivo. Furthermore, the cells at the edges of the slices are damaged by the slicing procedure. This initial amount of cell death was measured by TUNEL staining after a short ex vivo incubation and compared to later time points. Analyzing the fraction of TUNEL positive tumor nuclei by automated image analysis software is challenging, because tumor cell nuclei are in very close proximity in tumor tissue slices, precluding counting of individual nuclei . Therefore, we counted the number of DAPI-positive pixels that were also TUNEL positive in different image fields instead of analyzing numbers of nuclei (Fig. 3a, b). DAPI intercalates in the DNA and was regarded as an internal reference for total numbers of nuclei present. As a positive control, slices were incubated for 6 days with a high concentration of the chemotherapeutic drug Cisplatin (Fig. 3a, b). Some variation in initial cell death was noticed among different individual tumors. However, this initial amount of cell death did not increase drastically after prolonged ex vivo incubation (Fig. 3c).

### Ex vivo treatment of tumor slices with FAC chemotherapy

We investigated whether responses to the chemotherapy regimen FAC could be detected using our culture system and analytical methods. Because Cyclophosphamide demands metabolic activation, we used the pre-activated metabolite 4-HC (4-hydroperoxycyclophosphamide) for ex vivo experiments. Also, clinically used FAC chemotherapeutic drug concentrations and dosing schedules could not be translated directly into ex vivo treatments of tumor slices, therefore we performed our experiments with a single dose in which the slices were incubated for several days. In all experiments we retained a clinically used ratio of FAC components: 5-FU 500 mg/m^2^, doxorubicin 50 mg/m^2^, and cyclophosphamide 500 mg/m^2^, translating to a molar ratio of approximately 5-FU:Doxorubicin:4-HC = 46:1:22 [21]. To determine optimal dilution (dose) regimens for ex vivo experiments we tested several concentrations of this combination treatment with dilution #1 being the highest and dilution #10 being the lowest concentration (Table 2).

**Table 2:** Dose concentration of different FAC dilutions

	5’FU (46)^a^	Doxorubicin (1)^a^	4-HC (22)^a^
	(μM)	(μM)	(μM)
#1	460	10	220
#2	230	5	110
#3	115	2.5	55
#4	46	1	22
#5	23	0.5	11
#6	11.5	0.25	5.5
#7	4.6	0.1	2.2
#8	2.3	0.05	1.1
#9	1.15	0.025	0.55
#10	0.46	0.01	0.22
#11	0	0	0

^a^Indicates the molar concentration ratio for all different dilutions

Cell culture experiments revealed turning points for cytotoxicity between dilution #5 and #3 after three, four and five days of continuous exposure to FAC (data not shown). Hence, at least the negative control and the dilutions #7- #2 and incubation for three days were performed.

### Analysis of sensitivity based on tumor morphology

**Fig. 4:**
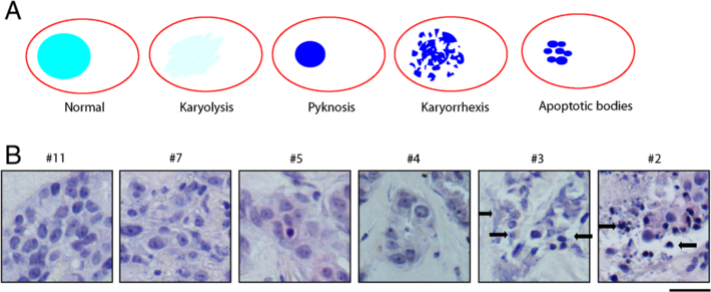
Assessment of drug response by aberrant nuclear morphology. **a** Nuclear morphology suggestive for tumor cell death included: Karyolysis: nuclear fading caused by dissolution of the chromatin, Pyknosis: irreversible condensation of the chromatin causing nuclei to shrink in size, Karyorrhexis: destructive fragmentation of a pyknotic nucleus, Apoptotic bodies: late stage apoptosis with fragmented nuclei. **b** An example of altered nuclear morphology (black arrows) observed in a single tumor after increasing dilution of FAC treatment. Dilution #3 was the lowest dilution at which altered nuclear morphology was observed in this tumor. Scale bar represents 50 μm

We first investigated whether the response to treatment was notable based on tumor cell morphology, which was assessed by H&E staining. The slides were scored for the presence of aberrant nuclei. Specific nuclear morphologies that were regarded as a marker of cell death included: karyolysis, pyknosis, karyorrhexis and apoptotic bodies (Fig. 4a). For each individual tumor we determined at which dilution the aberrant nuclear morphology appeared. That particular dilution was considered the threshold for tumor cell death (Fig. 4b). Five of the 15 tumors showed cell death at dilution #5, seven at dilution #3 and three at dilution #2. Histopathological features of these tumors did not correlate with the differences observed in drug responses (Table 3).

**Table 3:** Histo-pathological features of analyzed tumors

Sample	Histological subtype	Tumor size (cm)	B&R grade	Mitotic figures (per 2 mm^2^)	Receptor status			FAC response		
					ER	PR	HER2	Morpho-logy	EdU	TUNEL
059	ductal	3.4	2	9	+	-	-	5	*	5
068	ductal	1.5	1	2	+	+	-	3	5	3
070	lobular	4.0	2	2	+	+	-	5	5	5
071	lobular	5.5	2	1	+	+	-	5	5	5
072	ductal	11.0	2	2	+	+	-	2	2	2
083	ductal	6.5	2	2	+	+	-	5	7	5
089	ductal	3.8	3	16	+	+	-	3	7	5
090	ductal	3.4	3	13	+	-	-	2	3	2
102	lobular	7.0	2	7	+	+	-	2	5	5
112	ductal	3.5	3	15	+	+	+	3	3	3
118	ductal	1.7	3	12	+	+	-	3	3	5
119	ductal	7.0	3	26	-	-	-	5	3	3
121	lobular	9.7	2	6	+	+	-	3	5	3
135	ductal	5.5	3	16	+	+	-	3	3	3
141	ductal	2.5	3	8	+	-	-	3	5	5

B&R = Bloom and Richardson grade, ER = estrogen receptor, PR = progesterone receptor, Her2 = Her2/Neu receptor, FAC response: the dilution at which a threshold was observed. 1 being the highest FAC concentration and 10 being the lowest. * = data not available

### Analysis of sensitivity based on proliferation rate and cell death induction

**Fig. 5:**
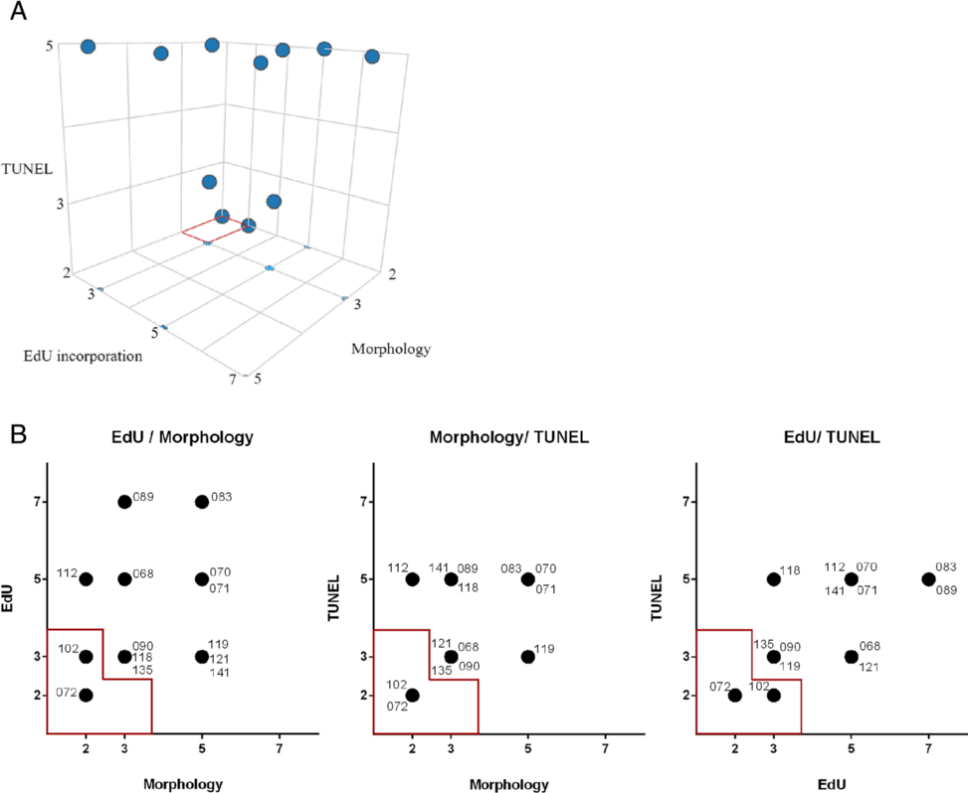
Analytical methods to asses cell proliferation and cell death in response to FAC treatment. **a** 3D representation of morphologic examination, EdU incorporation and TUNEL analysis per individual tumor. For every tumor the dilution at which a threshold was observed was plotted for each analytical method. Most sensitive tumors cluster in the upper most front part of the graph and most resistant tumors cluster at the lower back side of the 3D graph. Red box represents the resistant tumors based on arbitrary limits. **b** Scatterplots comparing two analytical methods for therapy response. For every tumor the dilution at which a threshold was observed was plotted for each analytical method. The resistant tumors based on arbitrary limits are outlined in red

We also analyzed the same 15 tumors for EdU incorporation and TUNEL staining and detected decrease in proliferation rate and induction of cell death after FAC treatment in the majority of tumors (Additional file 4: Figure S4 and Additional file 5: Figure S5). Arbitrarily, very sensitive tumors were defined as tumors displaying inhibited proliferation already at dilution #7, whereas resistant tumors where defined as tumors where proliferation was not inhibited up to dilution #3. Dilution #2 was found to be highly toxic as proliferation was completely absent in all tumors (Additional file 4: Figure S4, Additional file 6: Figure S6A, C). Similarly, cell death was observed at FAC concentrations as low as dilution #5 in more sensitive tumors, whereas more resistant tumors only displayed a clear induction of cell death at dilution #2 (Additional file 5: Figure S5, Additional file 6: Figure S6B, D). Comparison of the EdU and TUNEL measurements revealed that tumors 072 and 102 were always designated as most resistant regardless of the parameter measured. Also, assessment of sensitivity based on cell death detected by TUNEL or aberrant nuclear morphology showed great consistency in tumors (Fig. 5).

Among the 15 tumors there was one tumor derived from a patient who received neo-adjuvant FEC (5-FU, Epirubicin, Cyclophosphamide) treatment before surgical resection. The resected tumor was determined as having very little signs of treatment response by pathologists (Miller and Payne grade 2) [22]. In this particular tumor, cell death assessed by nuclear morphology and TUNEL assay was not induced until dilution #2 (Fig. 5, Additional file 6: Figure S6 and Table 3). This tumor also showed resistance when analyzed for EdU incorporation (M072, Additional file 6: Figure S6), suggesting that the ex vivo sensitivity assay can indeed identify resistant tumors.

## Discussion

We optimized the ex vivo culture system to preserve tumor morphology and tumor cell proliferation, while minimizing culture induced tumor cell death for up to seven days. We did not characterize later time points, but there are no signs of tissue deterioration after six days, suggesting that extended incubations may be possible if required for a specific functional assay. We specifically found that the culture of breast tumor slices is highly dependent on rotational movement during incubation, most probably by increasing nutrient exchange. Automated tissue slicing also increases ex vivo lifespan of tumor slices, as thinner slices allow better penetration of nutrients in the absence of active blood circulation. Finally, composition of the culture medium is highly important to maintain tumor slice viability with sufficient numbers of replicating cells for several days.

As traditional chemotherapies especially target cells in S phase or mitosis, tumor cell proliferation should be maintained during ex vivo culture of tissue slices to investigate chemotherapy responses. EdU incorporation is probably the most reliable biomarker for proliferation because it allows real time measurement of DNA synthesis in very short time intervals compared to other proliferation markers such as Ki-67, which remains positive for days after proliferation has ceased [23]. Cyclin A is a slightly better marker than Ki-67, but it also stains G2 phase cells and would therefore also mark cells that are blocked at the G2/M checkpoint. In our ex vivo tumor slice culture system active EdU incorporation is preserved for up to seven days, which is sufficient to detect most differences in drug response.

As decrease of EdU positive cells may be reversible when chemotherapy treatment has ended, more unidirectional response markers such as induction of cell death are important to define cytotoxic response to the given treatment. TUNEL staining is often used as a way of measuring cell death, although quantitative assessment of TUNEL staining remains a challenge. Measuring the number of individual TUNEL positive cells is hardly possible, because of the proximity of individual nuclei, which frequently even overlap in thin sections of organotypic BC slices. As an alternative option we determined the number of DAPI pixels that are TUNEL positive. The major advantage of this analytical method is that it can be performed automatically in a standardized high-throughput manner. This method may not be useful to distinguish subtle increases but it is suitable to detect major differences in TUNEL signal.

Similar culture methods have been used for ex vivo breast tumor slices in previous studies [15, 16, 20]. These report that viability and proliferation was retained for at least four to seven days. However, extensive validation of culture conditions for multiple tumors where not performed. Also, comparative analysis of different culture conditions is lacking from these reports. The tumor slice studies performed on head and neck, colon and lung tumors also lack some of the characterizations that have been performed in this study [6, 13]. Furthermore, the markers used as surrogates for treatment response, such as Ki-67, may not be the most adequate analysis for treatment response. Therefore, we propose to use EdU incorporation as the most direct and sensitive marker of proliferation in tumor tissue slices.

The responses to FAC treatment in our study were quantified based on morphologic examination, EdU incorporation and TUNEL assay. Interestingly, sensitivity classification based on the individual analytical methods did not differ significantly: the two least sensitive tumors always cluster together (Fig. 5). As morphologic examination and TUNEL assay both indicate tumor cell death, one of these methods would probably be sufficient, but more extensive characterization will be necessary to corroborate this conclusion.

Therapy resistance was arbitrarily defined in our cohort based on studies performed in BC patients receiving neo-adjuvant chemotherapy, which report that 10-20 % of primary breast tumors are resistant to treatment [24–26]. Interestingly, this arbitrarily defined threshold is consistent with the result obtained from one clinically proven therapy resistant tumor suggesting that our thresholds for therapy resistance may resemble the clinical outcome.

Actual clinical data regarding the response to FAC treatment are not yet available for our collected samples and it is therefore not yet possible to determine the predictive value of our ex vivo analysis method. Clinical responses to treatment will be monitored closely in the future in order to assess the predictive value of the ex vivo assay. However, this assessment may take years. Clinical data on the response to neo-adjuvant FEC treatment was only available for the patient having the least sensitive tumor in our analysis.

Ideally, the ex vivo analysis should be done with biopsies obtained prior to neo-adjuvant treatment, because this would enable determination of in vivo response of the tumor in a relatively short time frame. On the other hand, tumor heterogeneity may pose additional challenges to develop tumor biopsy based cytotoxicity assays into valid predictive tests. Moreover, the currently described methodology concerning this assay might not allow direct implementation of this assay in a clinical setting. Fresh tumor material is needed, timings are important and procedures are quite laborious. Therefore, automatization and high-throughput possibilities should be explored for this assay.

As this ex vivo assay does not take pharmacokinetics into account, it can only predict intrinsic sensitivity of tumor cells to a given treatment. In other words, an ex vivo resistant tumor is likely to also be resistant in vivo*, but* ex vivo sensitivity may not faithfully predict clinical sensitivity. Therefore, this cytotoxicity assay is primarily expected to be an effective tool to prevent unnecessary treatment of patients that harbor a therapy-resistant tumor, especially in advanced metastatic BC, where resistance to FAC chemotherapy is much more frequently observed than in primary BC. Similar assays can be developed to investigate other drugs, e.g. tamoxifen sensitivity.

## Conclusions

This study is the first to show extensive systematic optimization of breast tumor slices ex vivo by comparing different slicing techniques and culture conditions. Validation of optimal culture conditions was also systematically assessed by examining morphology, proliferation rate and cell death using robust markers. This culture system allowed detection of differences in tumor responses to FAC chemotherapy, which was confirmed by the observation that a clinically proven resistant tumor was identified in this way. The culture system and methods to analyze drug responses can be performed within a relatively short timeframe which makes it an effective tool to identify therapy-resistant tumors. This can prevent unnecessary treatment that would otherwise mostly cause side effects and a more promising treatment option can be started sooner.

### Ethics statement

Left over tumor tissue was used for research purposes according to the code of proper secondary use of human tissue in the Netherlands established by the Dutch Federation of Medical Scientific Societies and approved by the Erasmus MC Medical Ethical Committee (number MEC-2011-098).

### Consent statement

No informed consent was needed for this study. This was approved by the Erasmus MC Medical Ethical Committee (number MEC-2011-098).

## Additional files

Additional file 1: Figure S1. Title: Analysis of constant movement and optimal culture medium at 24 hours incubation. Description: Representative images of different tissue slices from one single tumor indicating microscopic fields harboring EdU positive cells. **A**. Manually sliced (approx. 2 mm) samples were incubated without constant movement conditions in different culture media. **B**. Without movement, the slices statically remained on the bottom of the culture dish during incubation. However, continuous movement caused slices to dynamically float in the culture medium which could increase nutrient exchange between the slice and the medium. **C**. Constant movement (60 rpm) significantly increased the number of EdU positive cells after 24 hours incubation compared to static culture conditions. Incubation in medium I resulted in the highest number of EdU positive cells compared to incubation with medium II, III and IV, in both static and constant movement conditions. Blue = DAPI, Red = EdU, Green lines indicate the edge of the tumor slice. (JPG 2671 kb) Additional file 2: Figure S2. Title: Analysis of constant movement and optimal culture medium at 48 hours incubation. Description: Representative images of different tissue slices from one single tumor indicating microscopic fields harboring the highest number of EdU positive cells. **A**. Control slices were directly labeled for two hours with EdU after slicing during constant movement at 60 rpm. **B**. Manually (approx. 2 mm) sliced samples were incubated without movement for 48 hours in different culture media. Very low numbers of EdU positive cells were present in the samples. Manually (approx. 2 mm) sliced samples that were incubated under constant movement conditions (60 rpm) for 48 hours in different culture media displayed higher number of EdU positive cells. **C**. Great heterogeneity in tumor stroma ratio was observed between different slices from the same tumor. **D**. Within the same slice also variation of proliferation indicated by EdU incorporation was noticed. This variation was partly explained by tumor-stroma variation but also by intrinsic variation in proliferation of tumor cells. (JPG 3683 kb) Additional file 3: Figure S3. Title: Morphology does not alter after prolonged incubation for 7 days. Description: Tumor slices from selected tumors were cultured for 7 days using optimized culture conditions. HE images represent slices from tumors incubated for indicated days. Morphologically no differences were noted between slices that were fixed on day 0 compared to slices fixed on day 7. Also, no differences were detected in tumor-stroma ratios after prolonged incubations. (JPG 2769 kb) Additional file 4: Figure S4. Title: Proliferation rate after incubation with FAC chemotherapy. Description: Tumor slices from each tumor where incubated with increasing concentrations of FAC. For EdU labeling samples were incubated with EdU for the last two hours of incubation. The numbers of EdU positive cells per mm2 keratin positive area where calculated. Sample M059 was not included in this analysis because no labeling was performed. Dilutions indicated with an asterisk (*) were missing because of insufficient tumor material. (JPG 787 kb) Additional file 5: Figure S5. Title: TUNEL assay after incubation with FAC chemotherapy. Description: Tumor slices from each tumor where incubated with increasing concentrations of FAC. TUNEL staining was performed for each tumor slice. Dilutions indicated with an asterisk (*) were missing because of insufficient tumor material. (JPG 1881 kb) Additional file 6: Figure S6.
**Title**: Differences in response to FAC chemotherapy in breast tumor slices. **Description**: Tumor slices were incubated in different increasing concentrations of FAC chemotherapy (Table 2). A. Tumor samples M072 and M083 display inhibition of proliferation at different doses of FAC. Proliferation is completely absent at dose #2. M072 stops proliferating at dose #3 whereas M083 already stops at dose #7. Blue = DAPI, Red = EdU. B. FAC induced cell death was detected by TUNEL assay. Massive cell death was induced at dose #2 for M072 and already at dose #5 for M083. Blue = DAPI, Green = TUNEL. C. Representation of heterogeneity in multiple analyzed images for proliferation. Black dots indicate median values of observed numbers of EdU positive cells per mm2 Cytokeratin area. Error bars indicate the interquartile range. D. Multiple image fields per tumor slice were analyzed for TUNEL signal. Black dots represent the median percentage of TUNEL-positive DAPI pixels. Error bars represent the interquartile range. (JPG 5390 kb)

## Abbreviations

BC: breast cancer
DAPI: 4',6-diamidino-2-phenylindole
EdU: 5-Ethynyl-2'-deoxyUridine
EU: 5-Ethynyl Uridine
FAC: 5-Fluororacil, Adriamycin, Cyclophosphamide
H&E: Haematoxilin & Eosin
PDX: patient derived xenograft
TUNEL: terminal deoxynucleotidyl transferase (dUTP) Nick End Labeling
