# Supplementary figures and images for: Tumor slice culture system to assess drug response of primary breast cancer
Source: BMC Cancer. 2016 Feb 9;16:78. doi: 10.1186/s12885-016-2119-2 (PMC4748539; doi:10.1186/s12885-016-2119-2)

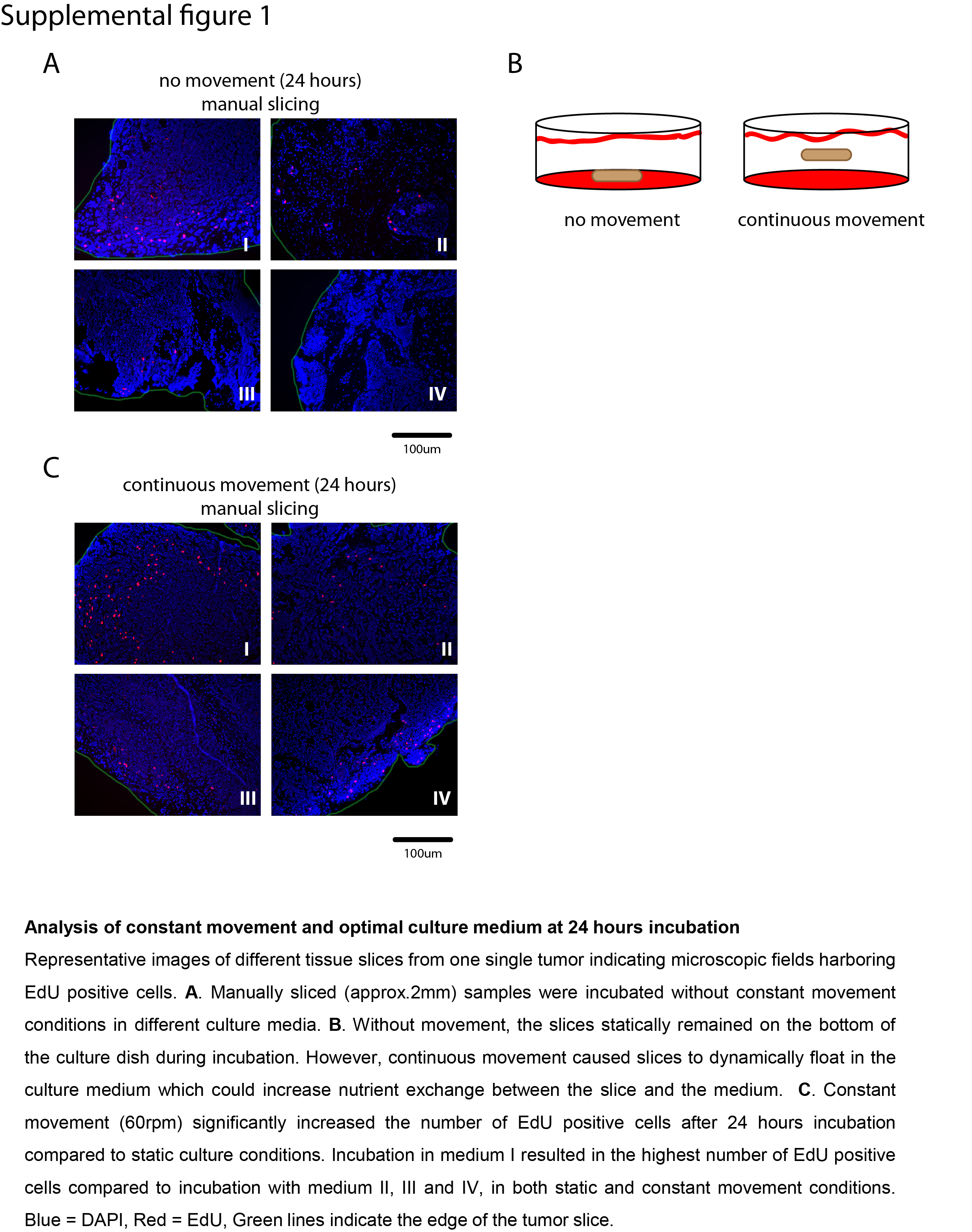

Supplement: Additional file 1: Figure S1. — Title: Analysis of constant movement and optimal culture medium at 24 hours incubation. Description: Representative images of different tissue slices from one single tumor indicating microscopic fields harboring EdU positive cells. A. Manually sliced (approx. 2 mm) samples were incubated without constant movement conditions in different culture media. B. Without movement, the slices statically remained on the bottom of the culture dish during incubation. However, continuous movement caused slices to dynamically float in the culture medium which could increase nutrient exchange between the slice and the medium. C. Constant movement (60 rpm) significantly increased the number of EdU positive cells after 24 hours incubation compared to static culture conditions. Incubation in medium I resulted in the highest number of EdU positive cells compared to incubation with medium II, III and IV, in both static and constant movement conditions. Blue = DAPI, Red = EdU, Green lines indicate the edge of the tumor slice. (JPG 2671 kb) [file 12885_2016_2119_MOESM1_ESM.jpg]

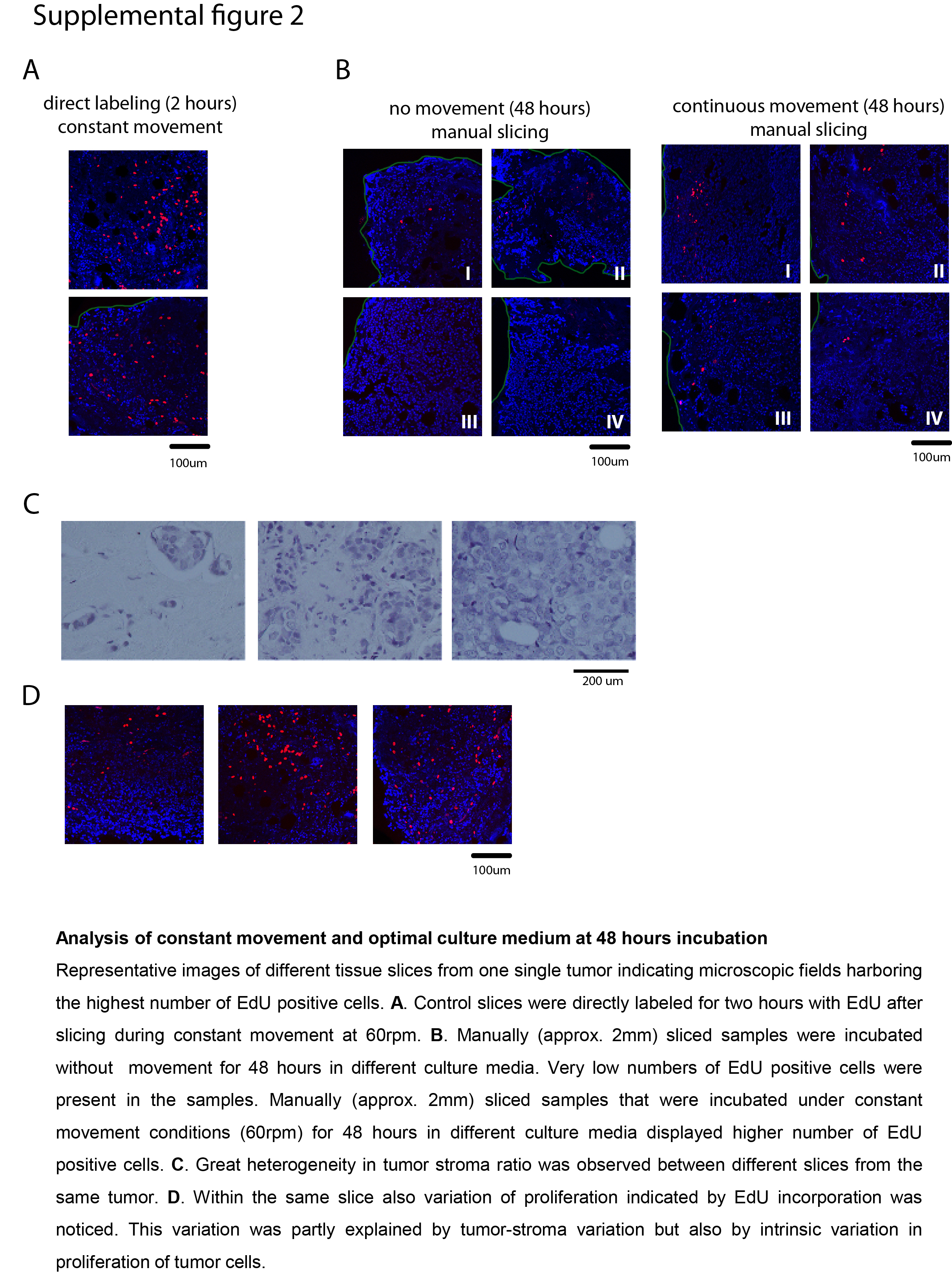

Supplement: Additional file 2: Figure S2. — Title: Analysis of constant movement and optimal culture medium at 48 hours incubation. Description: Representative images of different tissue slices from one single tumor indicating microscopic fields harboring the highest number of EdU positive cells. A. Control slices were directly labeled for two hours with EdU after slicing during constant movement at 60 rpm. B. Manually (approx. 2 mm) sliced samples were incubated without movement for 48 hours in different culture media. Very low numbers of EdU positive cells were present in the samples. Manually (approx. 2 mm) sliced samples that were incubated under constant movement conditions (60 rpm) for 48 hours in different culture media displayed higher number of EdU positive cells. C. Great heterogeneity in tumor stroma ratio was observed between different slices from the same tumor. D. Within the same slice also variation of proliferation indicated by EdU incorporation was noticed. This variation was partly explained by tumor-stroma variation but also by intrinsic variation in proliferation of tumor cells. (JPG 3683 kb) [file 12885_2016_2119_MOESM2_ESM.jpg]

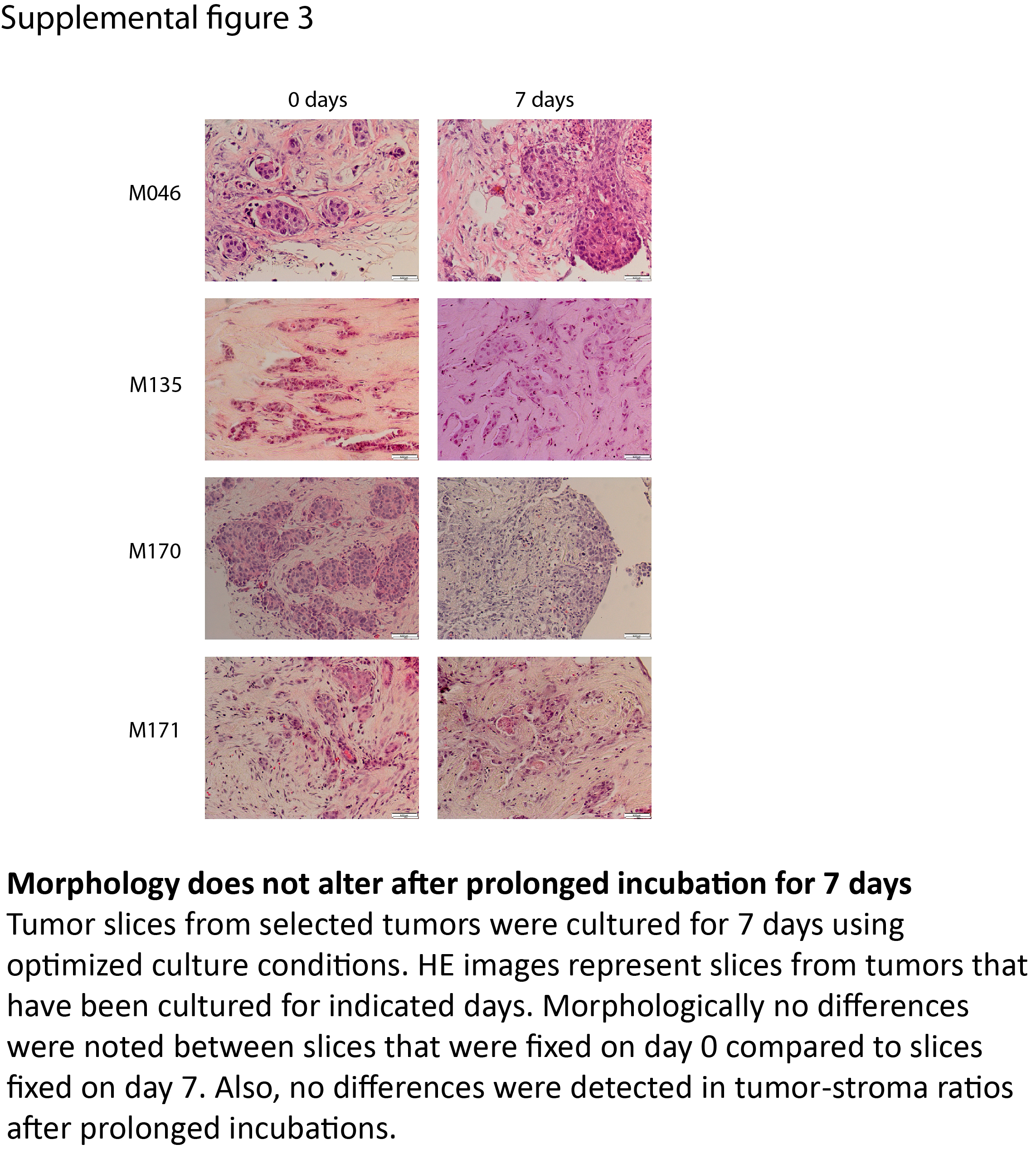

Supplement: Additional file 3: Figure S3. — Title: Morphology does not alter after prolonged incubation for 7 days. Description: Tumor slices from selected tumors were cultured for 7 days using optimized culture conditions. HE images represent slices from tumors incubated for indicated days. Morphologically no differences were noted between slices that were fixed on day 0 compared to slices fixed on day 7. Also, no differences were detected in tumor-stroma ratios after prolonged incubations. (JPG 2769 kb) [file 12885_2016_2119_MOESM3_ESM.jpg]

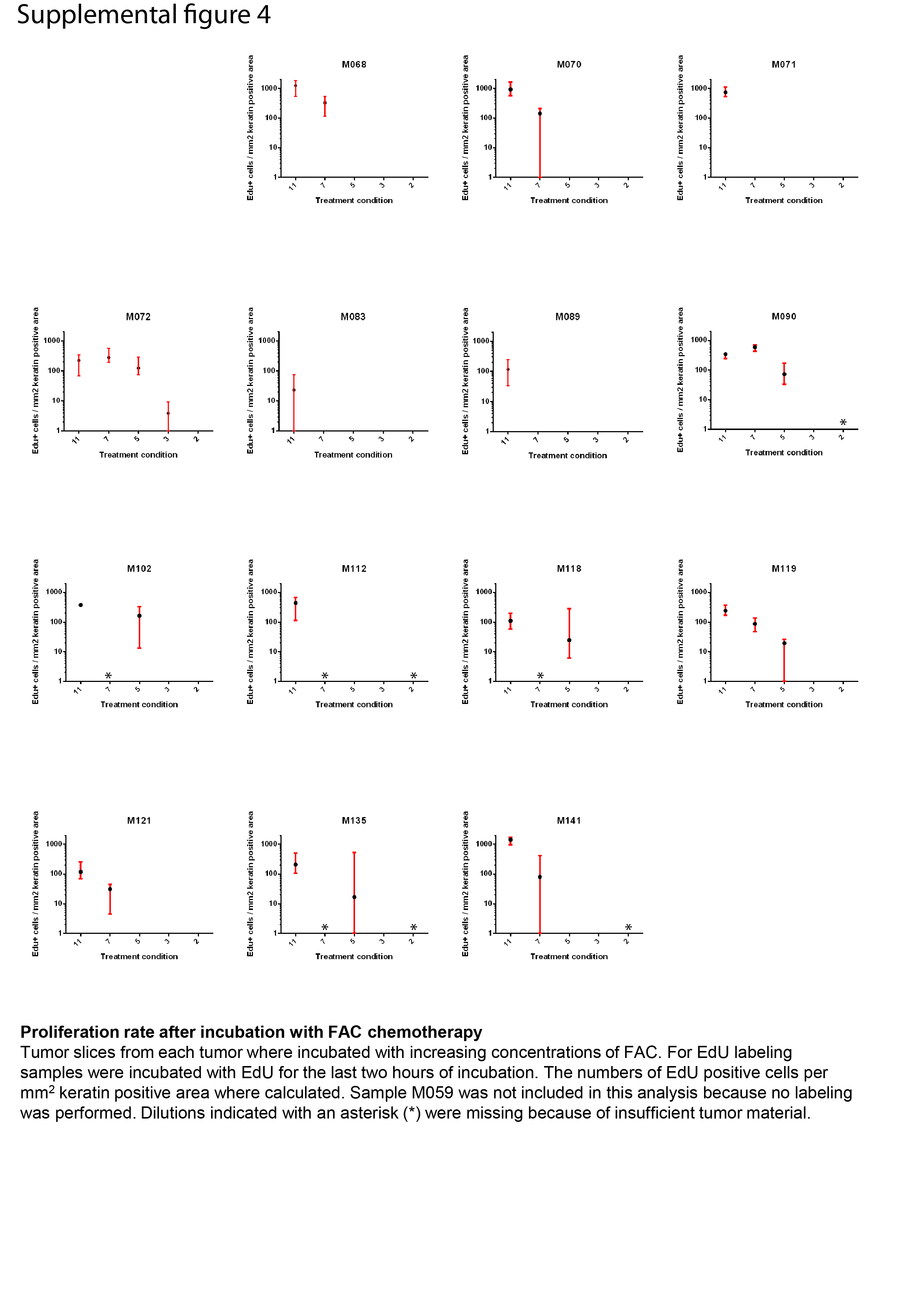

Supplement: Additional file 4: Figure S4. — Title: Proliferation rate after incubation with FAC chemotherapy. Description: Tumor slices from each tumor where incubated with increasing concentrations of FAC. For EdU labeling samples were incubated with EdU for the last two hours of incubation. The numbers of EdU positive cells per mm2 keratin positive area where calculated. Sample M059 was not included in this analysis because no labeling was performed. Dilutions indicated with an asterisk (*) were missing because of insufficient tumor material. (JPG 787 kb) [file 12885_2016_2119_MOESM4_ESM.jpg]

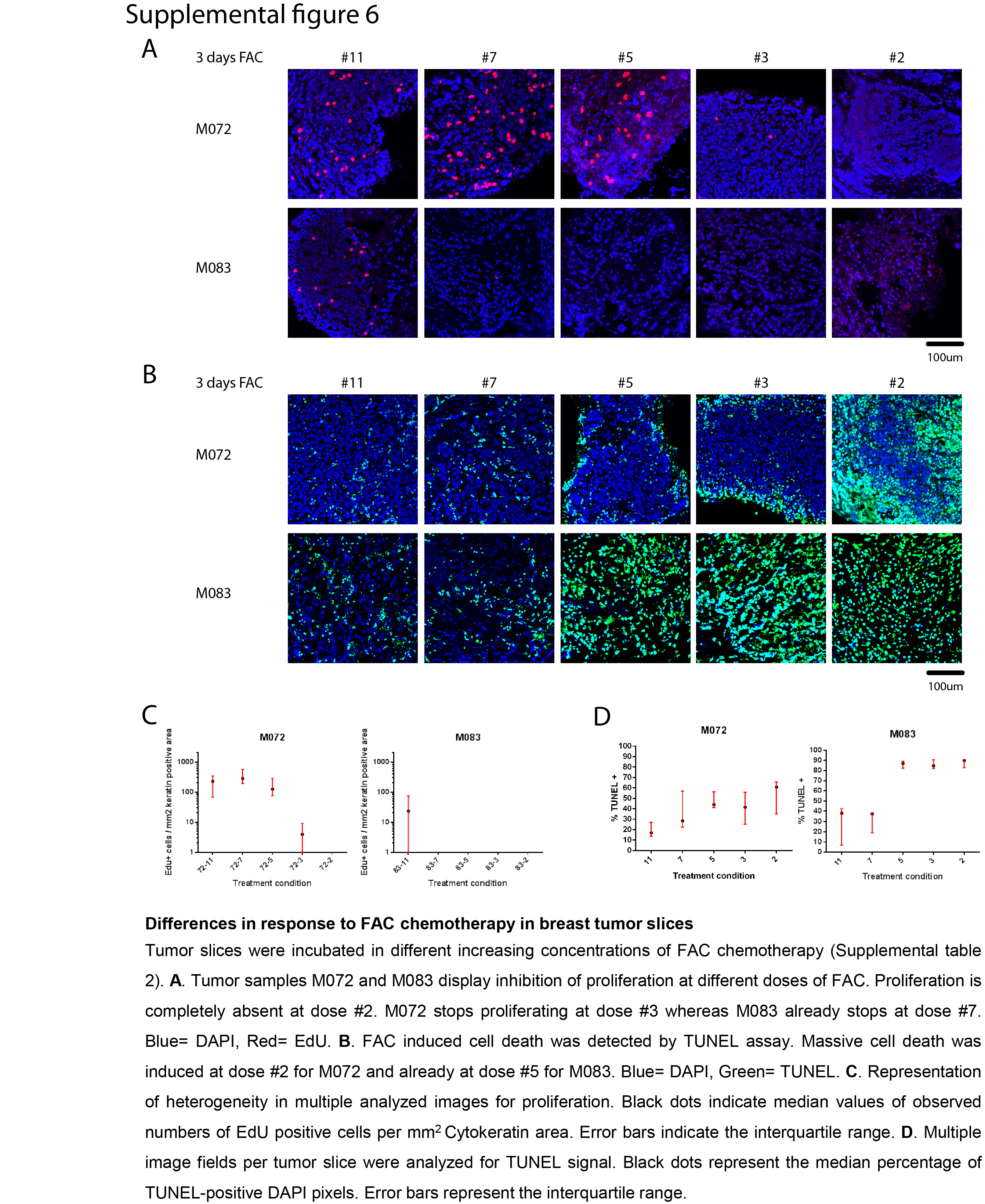

Supplement: Additional file 6: Figure S6. — Title: Differences in response to FAC chemotherapy in breast tumor slices. Description: Tumor slices were incubated in different increasing concentrations of FAC chemotherapy (Table 2). A. Tumor samples M072 and M083 display inhibition of proliferation at different doses of FAC. Proliferation is completely absent at dose #2. M072 stops proliferating at dose #3 whereas M083 already stops at dose #7. Blue = DAPI, Red = EdU. B. FAC induced cell death was detected by TUNEL assay. Massive cell death was induced at dose #2 for M072 and already at dose #5 for M083. Blue = DAPI, Green = TUNEL. C. Representation of heterogeneity in multiple analyzed images for proliferation. Black dots indicate median values of observed numbers of EdU positive cells per mm2 Cytokeratin area. Error bars indicate the interquartile range. D. Multiple image fields per tumor slice were analyzed for TUNEL signal. Black dots represent the median percentage of TUNEL-positive DAPI pixels. Error bars represent the interquartile range. (JPG 5390 kb) [file 12885_2016_2119_MOESM6_ESM.jpg]
